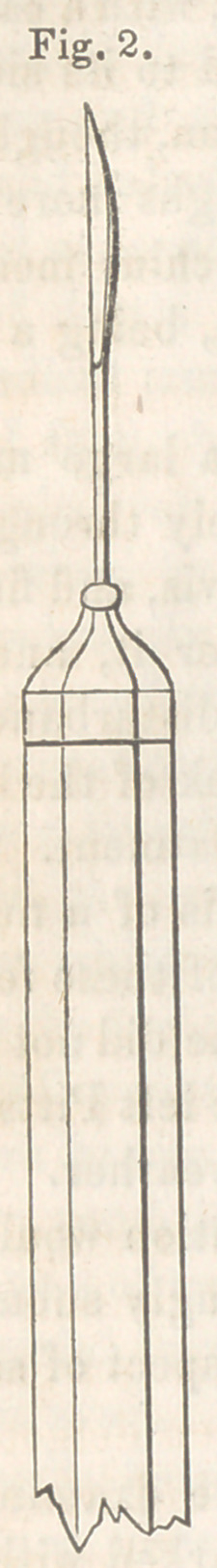# New Form of Cataract Needle

**Published:** 1855-06

**Authors:** John Neill

**Affiliations:** Professor of Surgery, Pennsylvania College


*New Form of Cataract Needle.* By John Neill, M.D., Professor of Surgery, Pennsylvania College.

In the operation for cataract by solution or absorption, every one has experienced the necessity of having an instrument that would not merely prick or perforate, but one that would also cut or divide the capsule and lens. In addition consequently to the old instruments recommended by Scarpa, Saunders and Adams, we have recently been favored with many improvements in this department of Surgery.

Fig. 1. Fig. 2.

Mr. Gemrig, the cutler, has lately made for me an instrument which I think combines all that is to be desired in a needle for this operation.

Figure 1 represents the real size and form of the needle, and figure 2 exhibits it enlarged two-fold.

The peculiarities are that it has but *one cutting edge*, which is *straight*, and it is therefore more readily sharpened, especially as *its sides* are *flat*, whereas in the double edged and lancet-shaped needles the edge is convex and the sides are also convex, and hence the difficulty in obtaining the great desideratum, a cutting edge. I have directed also that the edge of this miniature straight bistoury should form only one half of the needle, the remaining half or shank being cylindrical, so that it can be revolved readily in the wound through the tunics, which could not be done if the edge occupied the whole of the needle, as is the case in the old fashioned iris-knife.

The back of the needle is rounded and slightly convex, although this is not of much importance; but near the point the back should be sharpened slightly so as to facilitate its ready penetration. Its length is $\frac{5}{8}$ of an inch.

In a recent operation at the Pennsylvania Hospital, I had great satisfaction in the use of this needle, in dividing adhesions which had formed between the lens and iris, the result of inflammation subsequent to a previous operation.

I might also add that others engaged in this department of surgery, and whose experience and judgment I much value, have assured me of their satisfaction in its employment.

## Figures and Tables

**Fig. 1. f1:**
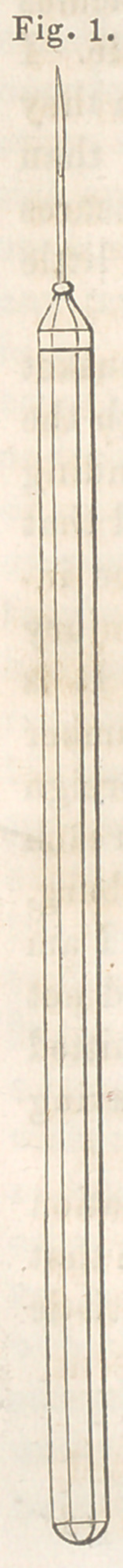


**Fig. 2. f2:**